# Utilization of Antimicrobial Agents in Intensive Care Units of Tertiary Care Hospital: An Observational Study

**DOI:** 10.31729/jnma.8936

**Published:** 2025-04-30

**Authors:** Sushant Aryal, Mili Joshi, Mayuri Gupta, Bipashwi Nath Uprety, Ram Krishna Shrestha, Pranita Shah, Piyush Rajbhandari

**Affiliations:** 1 Department of Pharmacology, Patan Academy of Health Sciences, Lagankhel, Lalitpur, Nepal; 2 Department of Microbiology, Patan Academy of Health Sciences, Lagankhel, Lalitpur, Nepal

**Keywords:** *antibiotic resistance*, *days of therapy*, *intensive care unit*, *Nepal*

## Abstract

**Introduction:**

The rising incidence of antibiotic resistance is a global threat. Monitoring antibiotic use is one of the strategies to address this issue. Intensive Care Units (ICUs) have high prevalence of antibiotic use and multidrug-resistant organisms. This study aims to study the status of antibiotic usage within the ICU in a tertiary care center.

**Methods:**

An observational cross-section study was conducted from July 15, 2021 to December 20, 2021 in the adult intensive care unit and record section of a tertiary care hospital of Nepal after approval from Institutional Review Committee. Data were collected from medical records of patients admitted to the ICU from January 1, 2018 to December 31, 2020 and who received antibiotics. Data were presented in terms of days of therapy per 1000 patient days (DOT/1000PD).

**Results:**

Total antibiotic consumption increased from 324.2 DOT/1000 PD in 2018 to 564.25 DOT/1000 PD in 2020. Consumption of Watch group antibiotic nearly doubled from 248.21 to 473.23 DOT/1000 PD, while Access antibiotics increased from 49.09 to 73.5 DOT/1000 PD. In contrast, Reserve group antibiotic usage declined from 26.91 to 17.49 DOT/1000 PD. In 2018, highest consumed antibiotics were piperacillin/tazobactam 57.89 (17.89%), azithromycin 53.98 (16.70%), ceftriaxone 49.58 (15.34%) and vancomycin 43.87 (13.57%) DOT/1000 PD. By 2020, their usage increased to 129.87 (23.02%), 69.38 (12.30%), 94.29 (16.71%), and 87.77 (15.55%) DOT/1000 PD respectively.

**Conclusions:**

Total antibiotic consumption increased over the years. There was a substantial rise in the use of Watch group antibiotics, particularly piperacillin/tazobactam, ceftriaxone, azithromycin, and vancomycin. Meanwhile, the use of Reserve group antibiotics declined during the same period.

## INTRODUCTION

Antimicrobial resistance (AMR) is a global health concern. In 2019, AMR was associated to around 5 million deaths and by 2050, is projected to cause 10 million deaths annually.^1^

The correlation between antibiotic use and the development of resistance is well-established.^2^ Inappropriate and excessive use in agriculture, humans and animals are major contributors to AMR, particularly in low-income countries like Nepal.^3,4^ Irrational prescriptions, dispensing antibiotics without prescriptions and self-medication are all associated with inappropriate antibiotic use in humans.^4^

Within the hospital, intensive care unit (ICU) has the highest prevalence of antibiotic use, much of which is inappropriate and has contributed to the development of multi-drug antimicrobial resistance.^5-8^ Days of therapy is suggested as a metric for measuring antimicrobial utilization in ICU.^9^ This study aims to study the status of antibiotic usage within the ICU in a tertiary care center.

## METHODS

An observational cross-section study was conducted at the Adult Intensive Care Unit (AICU) of a tertiary care hospital. The data was collected using the hospital records from July 15, 2021 to December 20, 2021. The ethical approval was obtained from Institutional Review Committee of Patan Academy of Health Sciences (REF: bss2105181525).

The study included the medical records of all the patients admitted in ICU from January 1, 2018, to December 31, 2020 and who had received antibiotics. Total population sampling was done. Regarding the antibiotics, those classified under the J01 category (antibiotics for systemic use) according to the Anatomical Therapeutic Chemical classification system were included.^10^ Medical records that were not found in the records section and those with missing data were excluded from the study. Since this study analyzed the past data, informed consent from each patient was not taken.

The investigator gathered data from the records section using a proforma. First, information on the total count of operational beds, ICU admissions, and the occupancy index was collected. Next, the unique identifier (hospital) number of ICU patients along with their admission and discharge dates were extracted. Medical records corresponding to these hospital number were then retrieved. Details about antibiotics, such as their names, routes of administration, frequency, number of doses, and duration of therapy were obtained from the medical records. Data was entered into Microsoft Excel 2003 and analyzed using the same software to calculate the Days of Therapy (DOT). This metric indicates the number of days a patient is administered an antimicrobial agent, irrespective of the dose.^9,11^ Any amount of an antimicrobial received within 24-hour period counts as 1 DOT. For a patient on multiple antibiotics, the total DOT is the sum of the DOTs for each individual antibiotic the patient is receiving.^11^ Antibiotic usage was then standardized per 1,000 patient days by dividing the DOT by the number of patient days and multiplying by 1,000. Patient days was determined by multiplying study duration (days) with bed strength and average bed occupancy rate. Data were summarized using frequencies and percentages.

## RESULTS

Out of 985 admitted patients, 630 medical records were included in the study. Over three years, the total days of therapy per 1000 patient days (DOT/1000 PD), was 324.20 in 2018, 446.45 in 2019, and 564.25 in 2020. Out of 324.20 DOT/1000 PD in 2018, penicillins had 66 (20.35%) DOT/1000 PD, cephalosporins had 61.7 (19.03%) DOT/1000 PD and macrolides had 54.7 (16.87%) DOT/1000 PD (Table 1).

**Table 1:** Annual antibiotic consumptions per antibiotic class in term of DOT per 1000 patients days (n=985).

	DOT per 1000 patient days			
Antibiotic class	2018	2019	2020	Overall (2018-2020)
	N (%)	N (%)	N (%)	N (%)
Aminoglycosides	5.87 (1.82)	13.05 (2.92)	6.82 (1.21)	25.74 (1.93)
Carbapenems	25.44 (7.87)	29.16 (6.53)	46.26 (8.20)	100.86 (7.56)
Cephalosporin				
1^st^ generation	0.49 (0.15)	0.96 (0.21)	0.59 (0.11)	2.04 (0.15)
3^rd^ generation	59.69 (18.47)	67.15 (15.04)	115.93 (20.55)	242.77 (18.19)
4^th^ generation	1.47 (0.45)	1.34 (0.30)	-	2.81 (0.21)
Fluoroquinolones	24.79 (7.67)	32.42 (7.26)	16.01 (2.84)	73.22 (5.49)
Glycopeptides	43.87 (13.57)	58.90 (13.19)	87.77 (15.55)	190.53 (14.28)
Macrolides	54.63 (16.90)	70.41 (15.77)	74.72 (13.24)	199.76 (14.97)
Penicillins				
Non-anti Pseudomonal	8.15 (2.52)	13.05 (2.92)	15.71 (2.79)	36.92 (2.77)
Antipseudomonal	57.89 (17.86)	87.10 (19.51)	129.87 (23.02)	274.87 (20.59)
Miscellaneous	41.91 (12.97)	72.91 (16.33)	70.57 (12.51)	185.39 (13.89)
Total	323.20 (100)	446.45 (100)	564.25 (100)	1334.90 (100)
DOT: Days of therapy				

Similarly, Out of 324.20 DOT/1000 PD, piperacillin/tazobactam had 57.89 (17.86%) DOT/1000 PD, azithromycin had 53.98 (16.70%) DOT/1000 PD, ceftriaxone 49.58 had (15.34%) DOT/1000 PD and vancomycin had 43.87 (13.57%) DOT/1000 PD (Table 2).

**Table 2:** Annual antibiotic consumptions in term of DOT per 1000 patients days (n=985).

Antibiotic	DOT per 1000 patient days			
	2018	2019	2020	Overall (2018-2020)
	N (%)	N (%)	N (%)	N (%)
Piperacillin/Tazobactam	57.89 (17.86)	87.10 (19.51)	129.87 (23.02)	274.87 (20.59)
Ceftriaxone	49.58 (15.34)	60.63 (13.58)	94.29 (16.71)	204.49 (15.32)
Azithromycin	53.98 (16.70)	67.92 (15.21)	69.38 (12.30)	191.28 (14.33)
Vancomycin	43.87 (13.57)	58.90 (13.19)	87.77 (15.55)	190.53 (14.27)
Levofloxacin	19.24 (5.95)	27.05 (6.06)	14.83 (2.63)	61.12 (4.58)
Imipenem	22.50 (6.96)	20.91 (4.68)	10.67 (1.89)	54.09 (4.05)
Meropenem	2.94 (0.91)	8.25 (1.85)	35.58 (6.31)	46.77 (3.50)
Metronidazole	11.90 (3.68)	18.23 (4.08)	16.01 (2.84)	46.14 (3.46)
Doxycycline	10.76 (3.33)	14.77 (3.31)	18.38 (3.26)	43.92 (3.29)
Co-trimoxazole	5.71 (1.77)	14.01 (3.14)	13.64 (2.42)	33.35 (2.50)
Cefotaxime	5.71 (1.77)	5.95 (1.33)	15.42 (2.73)	27.07 (2.03)
Rifaximin	2.94 (0.91)	10.17 (2.28)	13.34 (2.36)	26.45 (1.98)
Amikacin	4.73 (1.46)	8.83 (1.98)	6.82 (1.21)	20.37 (1.53)
Clindamycin	6.20 (1.92)	5.18 (1.16)	2.37 (0.42)	13.75 (1.03)
Amoxicillin/Clavulanate	2.45 (0.76)	2.69 (0.06)	7.12 (1.26)	12.25 (0.92)
Linezolid	3.42 (1.06)	6.72 (1.50)	2.08 (0.37)	12.22 (0.92)
Ciprofloxacin	5.54 (1.72)	5.37 (1.20)	1.19 (0.21)	12.10 (0.910
Ampicillin	2.61 (0.81)	3.26 (0.73)	4.15 (0.74)	10.02 (0.75)
Colistin	0.98 (0.30)	3.84 (0.86)	4.74 (0.84)	9.56 (0.72)
Cefixime	3.26 (1.01)	0.19 (0.04)	5.34 (0.95)	8.79 (0.66)
Clarithromycin	0.65 (0.20)	2.49 (0.56)	5.34 (0.95)	8.48 (0.64)
Cloxacillin	1.96 (0.61)	2.88 (0.64)	1.48 (0.26)	6.32 (0.47)
Amoxicillin	1.14 (0.35)	0.77 (0.17)	2.97 (0.53)	4.87 (0.37)
Gentamicin	1.14 (0.35)	2.69 (0.60)	-	3.83 (0.29)
Flucloxacillin	-	3.45 (0.77)	-	3.45 (0.26)
Cefepime	1.47 (0.45)	1.34 (0.30)	-	2.81 (0.21)
Cefazolin	0.49 (0.15)	0.96 (0.21)	0.59 (0.11)	2.04 (0.15)
Streptomycin	-	1.53 (0.34)	-	1.53 (0.11)
Ceftazidime	-	0.38 (0.09)	0.89 (0.16)	1.27 (0.10)
Cefpodoxime	1.14 (0.35)	-	-	1.14 (0.09)
Total	324.20 (100)	446.45 (100)	564.25 (100)	1334.90 (100)
DOT: Days of therapy				

Out of 30 antibiotics used, 12 (40%) were from the Access group, 15 (50%) from the Watch group, and 3 (10%) from the Reserve group. In 2018, antibiotics from the Watch group were prescribed 14 (51.85%) times, Access group 11 (40.74%) times, and Reserve group 2 (7.41%) times. In 2019, Watch group prescriptions were 15 (51.72%), Access group 12 (41.38%), and Reserve group 2 (6.90%). In 2020, Watch group prescriptions were 13 (52%), Access group 10 (40%), and Reserve group 2 (8%).

**Table 3:** Annual antibiotic consumption by AWaRe group in term of DOT per 1000 patients days (n=985).

AWaRe			
	2018	2019	2020
	N (%)	N (%)	N (%)
Access	49.09 (15.14)	77.70 (17.40)	73.53 (13.03)
Watch	248.21 (76.56)	337.29 (75.54)	473.23 (83.87)
Reserve	26.91 (8.30)	31.46 (7.05)	17.49 (3.10)
Total	324.20 (100)	446.45 (100)	564.25 (100)
DOT: Days of therapy			

Out of 34 antibiotic, 23 (76.7%) of them were from the World Health Organization Essential Medicines List (EML), 22 list, 2021 and 24 (80%) were from the National EML, 6th revision, 2021. Additionally, 27 (90%) of the drugs were prescribed by their generic names.

## DISCUSSION

Our findings showed increase in antibiotic consumption from 324.2 days of therapy per 1000 patient days (DOT/1000 PD) in 2018 to 564.25 in 2020. Several reasons likely contributed to this trend. Firstly, infections are a leading cause for admission and treatment in ICU.^5-8^ The rising incidence of severe bacterial infections, including multidrug-resistant organisms in low-income country like Nepal could have an increased need for antibiotic treatment.^12^ Secondly, timely initiation of antibiotic therapy is essential for recovery of patient in ICU. However, determining the appropriate timing is challenging due to the difficulty in identifying whether a patient's symptoms are caused by a bacterial infection or another condition. This uncertainty may lead to the overuse of antibiotics.^6-8^ Additionally, the Coronavirus disease 2019 (COVID-19) pandemic may have influenced antibiotic usage practice. Early in the pandemic, healthcare systems faced challenges in managing COVID-19 because of the absence of standardized treatment protocols which led to many different approaches. Despite the disease being viral, antibiotics were also frequently used. This could have also influenced physician to prescribe antibiotic in non-COVID-19 ICU patients as well.^12^ Finally, inadequate monitoring of antibiotics in ICU can also contribute to their over-prescription and misuse. Research indicates 70% of ICU patients receive antibiotic of which 30-60% are unnecessary.^13^

Our study showed that the antibiotics with high usage in term of D0T/1000 PD were piperacillin/tazobactam 274.87 (20.59%), ceftriaxone 204.49 (15.32%), azithromycin 191.28 (14.33%), and vancomycin 190.53 (14.27%). This antibiotic usage pattern was comparable to the findings from Candeloro et al. study, which also reported high usage of piperacillin/tazobactam 296.25 (21.6%), and vancomycin 187.2 (13.7%) However, the study by Candeloro et al. showed a higher usage of carbapenems 196.3 (14.3%) and cefazolin 130.86 (9.6%), while the use of azithromycin was lower 36.35 (2.7%).^14^ In contrast, our study found a lower usage of carbapenems 100.79 (7.56%) and cefazolin 2.04 (0.15%). When comparing our findings to those of Balkhy et al., use of piperacillin/tazobactam 145.9 and vancomycin 129.5 was lower while the use of carbapenems 235.7 was higher.^15^ In a study by De Bus et al., most frequently prescribed antibiotic was anti-pseudomonal penicillins/beta-lactamase inhibitor 218.2. Use of third generation cephalosporin was 41.2, macrolides was 30 and glycopeptides was 62.6. 9 Furthermore, various studies conducted in Nepal to assess prescription patterns in ICUs found that piperacillin/tazobactam, ceftriaxone, and azithromycin were commonly prescribed antibiotics.^16, 17^

In critical care settings, healthcare providers often treat seriously ill patients with unknown sources of infection and causative agent. Because of this, doctors typically start with broad-spectrum antibiotics that cover a wide range of potential pathogens until more specific information becomes available.^6-8^ The possible reason for high use of piperacillin/tazobactam could be because of its broad-spectrum activity. It is effective against a wide range of bacteria, including many gram-negative and gram-positive pathogens. This makes it the preferred choice for treating critically ill patients that are admitted in ICU. Similarly, ceftriaxone, a third-generation cephalosporin, is also preferred because of its high potency, broad spectrum of activity, convenient once or twice daily dosing and the low incidence of adverse drug reactions. Additionally, azithromycin is frequently used in the ICU for its effectiveness against atypical respiratory pathogens like Chlamydia pneumoniae and Mycoplasma pneumoniae, which are common causes of atypical pneumonia in ICU patients. However, broad spectrum qualities of these drugs may also contribute to frequent misuse.^18^ Moreover, it is a common practice in Nepal for individuals to take antibiotics without a prescription.^18^ This practice of self medication increased during the COVID-19 pandemic because of limited knowledge and the fear of disease.^12^ By the time they visit hospital for the treatment; many patients would have already used different types of antibiotics on their own and are often severely ill.^12, 19^ This could be the another reason of high use of broad-spectrum antibiotics.

Prasil et. Al. has reported the rise in methicillin resistance staphylococcus aureus prevalence and its 100% sensitivity to vancomycin.^20^ This finding indicates that vancomycin remains a highly effective treatment against MRSA infections, which may explain its increased usage in our study. However, studies have also found inappropriate use of vancomycin in ICU setting, often due to its frequent empiric use for suspected life-threatening Gram-positive infections.^21^

The World Health Organization (WHO) Expert Committee on Selection and Use of Essential Medicines developed the Access, Watch, and Reserve classification of antibiotics to monitor antibiotic usage and support antimicrobial stewardship. This classification suggests that at least 60% of antibiotic consumption should be from the Access group.^22^ In our study, use of Access group antibiotic was below 60%. Days of therapy per 1000 patient days (DOT/1000 PD) for Watch group antibiotics increased from 248.2 days in 2018 to 473.2 days in 2020. Conversely, the DOT of Reserve group antibiotics declined from 26.9 daysin 2018 to 17.5 days in 2020. "Access" antibiotics are intended for empiric treatment of common infections, "Watch" antibiotics are used for more severe conditions, and "Reserve" antibiotics are reserved for critical cases involving severe infections caused by multidrug-resistant pathogens.^22^ In ICU settings, patients often are critically ill and infections caused by MDR pathogens are common, this might be the possible reason of frequent use of Watch and Reserve group antibiotics observed in this study. ^5 6^

In Nepal, drugs are commonly prescribed in brand names rather than generic names because of lack of policies for generic prescribing and substitution, unlike developed countries.^23^ There are also concern regarding the interchangeability and quality assurance of generic medicines as well as inadequate awareness about it among healthcare professionals.^23^ However, prescribing in generic name has several advantages such as cost effectiveness and reduces confusion among doctors, pharmacist and patient.^23^ In our study, the majority of prescribed drugs 27 (90%) were in generic form. The study revealed that 23 (76.6%) of prescribed antimicrobials were from the WHO EML, 22 list, 2021 and 80% were from the National EML, 6th revision, 2021. and 24 (80%) were from the National EML.^24, 25^ High uses of antibiotic from EML indicate that efficacious, safe, easily available and cost-effective drug were used.^25^

To our knowledge, this is the first study in the country to measure antibiotic utilization in ICUs using DOT. DOT was selected because it measures total days of antibiotic therapy regardless of dose strength or frequency which is important in ICU settings where critically ill patients often require doses that differ from standard recommendations.

Limitation of study was that the data were collected from paper-based medical records which increased the chances of missing medical records and information. Additionally, we did not assess whether the antibiotic use was rational, if guidelines were followed, whether treatment was empirical or directed, or if it started in the ICU or beforehand. Moreover, there were few articles globally or nationally that used DOT as the metric for utilization making it challenging to compare our finding.

Several solutions are recommended. Firstly, switching to electronic medical records would reduce the risk of missing data and ensure complete information is collected. Secondly, strengthening infection control measures in ICUs can prevent hospital infections, thereby reducing the necessity for antibiotic use. 26 Additionally, establishing antibiotic stewardship programs in ICUs will further ensure appropriate antibiotic use and adherence to guidelines.^6-8^

## CONCLUSIONS

Overall antibiotic consumption increased, with the highest usage observed in Watch group antibiotics. There was a slight increase in usage of Access group antibiotic while the use of Reserve group declined. Piperacillin/tazobactam, ceftriaxone, azithromycin, and vancomycin were the most commonly consumed antibiotic. Additionally, most antibiotic were prescribed by their generic names and were on the National Essential Drug List (6th revision, 2021). Improving infection control measures and antibiotic stewardship in ICU could help reduce antibiotic use.
